# Plasma–Saline Water Interaction: A Systematic Review

**DOI:** 10.3390/ma15144854

**Published:** 2022-07-12

**Authors:** Tatiane Fonseca de Melo, Lucas Cabral Rocha, Rútilo Pereira Silva, Rodrigo Sávio Pessoa, Andreia Mitsa Paiva Negreiros, Rui Sales Júnior, Moisés Bento Tavares, Clodomiro Alves Junior

**Affiliations:** 1 Laboratorio de Plasma Aplicação na Agricultura, Departamento de Ciências Exatas e Naturais, Saúde e Meio Ambiente—Labplasma, Universidade Federal Rural do Semiárido, Mossoró 59625-900, Brazil; lucas.rocha48397@alunos.ufersa.edu.br (L.C.R.); teluufersa2017@gmail.com (R.P.S.); clodomiro.jr@ufersa.edu.br (C.A.J.); 2 Laboratório de Plasmas e Processos, Instituto Tecnológico de Aeronáutica, São José dos Campos 12228-900, Brazil; rspessoa@ita.br; 3 Departamento de Ciências Agronômicas e Florestais, Universidade Federal Rural do Semi-Árido, Mossoro 59625-900, Brazil; andreiamitsa@gmail.com (A.M.P.N.); ruisales@ufersa.edu.br (R.S.J.); sestavaresagro@gmail.com (M.B.T.); 4 Programa de Pós-Graduação em Engenharia Mecânica, Universidade Federal do Rio Grande do Norte, Natal 59078-970, Brazil

**Keywords:** saline water, atmospheric plasma, corona discharge, DBD, PRISMA

## Abstract

Plasma–liquid interaction research has developed substantially in recent years due, mostly, to the numerous applications of cold atmospheric plasma (CAP). Plasma–liquid interactions are influenced by the concentrations of the ionic species present in the liquid environment, and few studies have paid attention to saline water, which generally mediates the reactions in many plasma applications. Therefore, the present review aims to explore the main results and the influence of variables on the modification of properties of saline water by CAP sources following the guidelines of the Preferred Reporting Items for Systematic Reviews and Meta-Analyses (PRISMA). The searches were carried out in the Scopus, Science Direct, and Web of Science databases, resulting in the inclusion of 37 studies. The main effects of the interaction between CAP and saline water are (i) the production of reactive oxygen and nitrogen species (RONS); (ii) the increase in conductivity and decrease in pH, directly proportional to the increase in discharge voltage; (iii) and the effective area of interaction and the shortest distance between electrode and solution. Other effects are the localized evaporation and crystallization of salts, which make the interaction between plasma and saline water a promising field in the development of technologies for desalination and improvement of liquid properties.

## 1. Introduction

Although investigations of plasma–liquid interaction began more than 100 years ago through high-energy pulse systems or high-voltage isolation [1,2], it was only at the beginning of this century that substantial growth in this research was observed. This is mainly due to the numerous applications of cold atmospheric plasma (CAP), including in liquid media [3,4]. The liquid–CAP interaction is being increasingly studied due to its importance in applications such as nanoparticle (NP) synthesis [3,5], environmental remediation [6], sterilization [7], agriculture [8], biology [9], medicine [10,11,12,13], and food [14,15,16]. These and other applications are possible due to the generation of UV radiation, shock waves, and active radicals from the plasma, whose reaction product with the liquid is, almost always, the precursor of the modification of material properties [17,18].

When plasma touches the surface of saline water, the reactive species interact with the solvated charges in the saline water and induce additional physicochemical transformations, having as one of the consequences the accelerated and selective crystallization of salts, for example, which opens up possibilities for use in desalination processes, chemical extraction, and others [19]. To date, the effects of the interaction of plasma with saline water have been reported for sterilization and disinfection [20,21,22,23]; desalination, precipitation, and crystallization of salts [19,24,25,26]; catalysis of oxidative processes [27,28,29]; decontamination of organic pollutants [30]; alternative sources of nitrites/nitrates [31,32]; etc. There are different applications and different types of plasma sources, which differ strongly from each other, making it difficult to summarize or even compare the results of different types of sources from a chemical and physical point of view.

Another important point to consider is the form of energy distribution among the plasma species. If the kinetic energy of the electrons (Te) is much greater than the kinetic energy of the other species (Tg), we can say that this is a CAP. If the electronic temperature is approximately equal to the temperature of the other plasma species, it approaches the local thermal equilibrium (LTE), which is called hot atmospheric plasma (HAP). While in HAP plasma transitions and chemical reactions are carried out by collisions, in CAP these events are governed by radioactive processes [33].

Most plasma systems deviate from local equilibria. What differentiate them are, mainly, the means of excitation of the power sources used. Depending on the excitation frequency, the electronic collision rate and thus the reaction kinetics of the plasma–liquid interaction will also vary. Generally speaking, they can be produced by (i) pulsed corona discharge, when high voltage pulses are applied between a thin conducting wire and a plane; (ii) dielectric barrier discharge (DBD), when the potential is applied between two electrodes separated by dielectrics, and (iii) a plasma jet, which consists of the application of high-frequency voltage between two coaxial electrodes, between which a gas feedstock (usually inert gas or mixtures with other gases) flows at a high rate. While the first two systems produce the active species directly on the surface to be processed, in the plasma jet system, part of the species are produced in the jet nozzle and carried by the carrier gas to the surface to be processed. That is, if the same type and quantity of species is produced in this last system as in the pulsed corona, for example, the plasma jet system will be less reactive. On the other hand, if a corona system with a DBD that has the same electrical power are compared, it is very likely that the DBD will be more effective due to the greater distribution of the discharge over the processed surface. It is clear, therefore, that process parameters such as power, voltage, frequency of the applied electric field, pulse width, treatment time, effective area of interaction, atmosphere composition, and the saline composition of the water effectively influence the results of interaction with the plasma [34,35,36]. Identifying how these parameters influence the plasma–salt water interaction and how to correlate them is not an easy task but is necessary when we want to compare the different processes.

The present systematic review aims to understand how plasma parameters influence plasma–saline water interactions, as well as the correlation between them, in the results available in the literature. So far, the authors are not aware of the existence of reviews in this specific sense, and a study of the interaction of plasma with saline waters is very opportune. It can contribute to advances around water desalination or even saline effluent recovery, in addition to its importance in applications in health, agriculture, and the environment.

## 2. Materials and Methods

This systematic review was carried out in accordance with the recommendations of the items of the Preferred Reporting Items for Systematic Reviews and Meta-Analyses (PRISMA) [37].

### 2.1. Databases and Search Strategy

The article searches were carried out in the Scopus, Science Direct and Web of Science databases from 25 August to 1 December 2021 using the following keyword combinations: plasma andseawater and desalination; plasma and brine; plasma and “saline water”; plasma and bittern; “electric discharge” and desalination; “electric discharge” and seawater; “electric discharge” and brine. In all searches, only filters for the year of publication were used.

**Figure 1:**
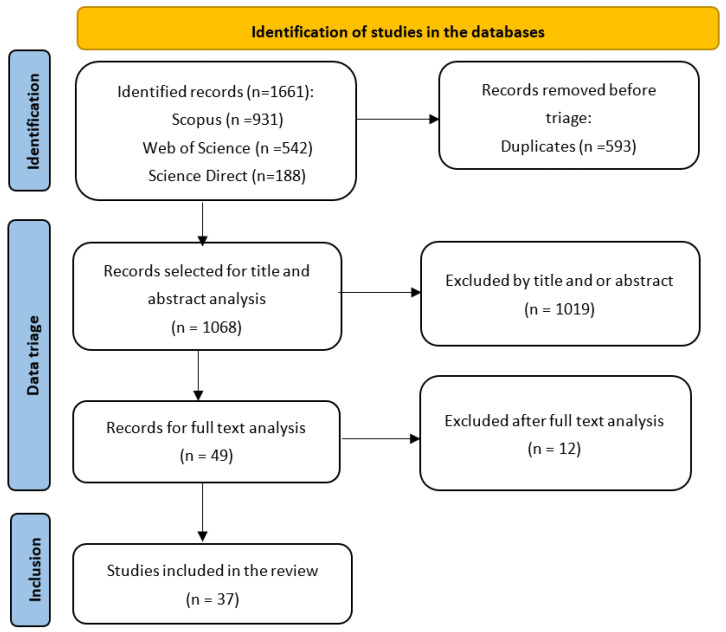
Selection flowchart of studies to be reviewed.

Search results were exported to the Rayyan management tool (https://www.rayyan.ai/, accessed on 14 May 2022). After removing duplicate articles, a first selection of studies was performed by analyzing the title and abstract. The pre-selected works at this stage underwent full text analysis for later inclusion or definitive exclusion (Figure 1).

### 2.2. Eligibility Criteria

The following criteria were used for the inclusion of articles: (1) to report and discuss the application, physical and chemical reactions triggered by the plasma–saline water interaction; (2) publications from 2000 to 2021; (3) English language publications; and (4) original research studies. Review articles, books, book chapters, theses, letters to the editor, and conferences were excluded, as well as other studies that did not meet the aforementioned inclusion criteria.

### 2.3. Data Extraction and Evaluation of Studies

A form was prepared to extract the data of interest from the included studies, namely, name of authors, year of publication, objective, characteristics of the treated saline water, parameters of the plasma treatment used, and main responses observed experimentally.

Using this information, the evaluation of the studies was performed by tabulating, managing, and comparing data regarding the main responses observed for plasma configurations in different plasma–saline water interactions.

## 3. Results and Discussion

Of the 1661 results obtained from the databases, after removing the duplicate files, 1068 studies were analyzed. Through the eligibility criteria, a total of 37 articles remained (Table 1). Although there were different objectives, they all converged regarding the results on the plasma–salt solution interaction.

Another 12 potentially eligible studies were excluded for the following reasons: conference publication (1 article); publication in a language other than English (1 article); and not presenting results and discussions directly related to the physical and chemical reactions of the plasma saline water interaction (10 articles).

### 3.1. Treatment Parameters

**Figure 2:**
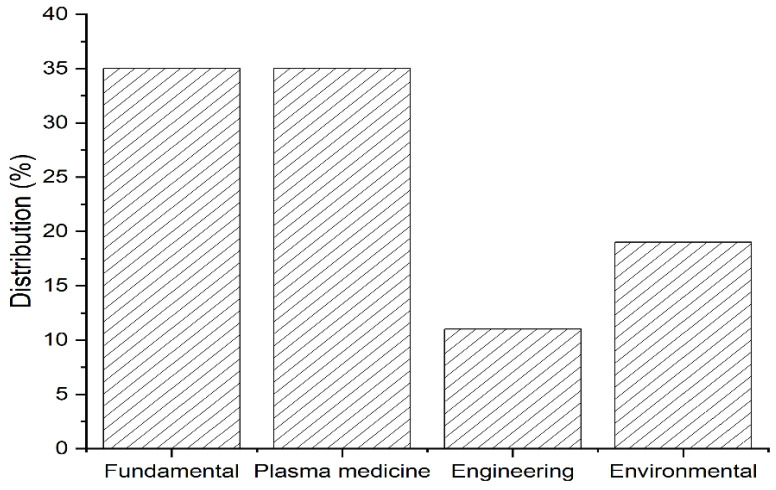
Macro-research fields of the included studies in this review. Fundamental [21,41,42,43,44,45,46,47,50,52,54,58,59], plasma medicine [20,22,31,32,38,39,40,48,49,51,55,56,60], engineering [24,25,26,53], and environmental [19,23,27,28,29,30,57].

Of the 37 articles chosen, most of the investigations do not apply to an isolated research field, but as associations between environmental and biological, physical or chemical studies, for example [20,21,22,23,29,38,50,52]. However, considering the main parameters investigated by the authors and the research objectives, a distribution of studies in macro-research fields allows a more detailed visualization of the main sectors in which the treatment is currently used (Figure 2). The works in the fundamental area and plasma medicine correspond to 70% of the total, with 35% for each research field. In addition to these, there are works in the environmental (19%) and engineering (11%) area.

**Figure 3:**
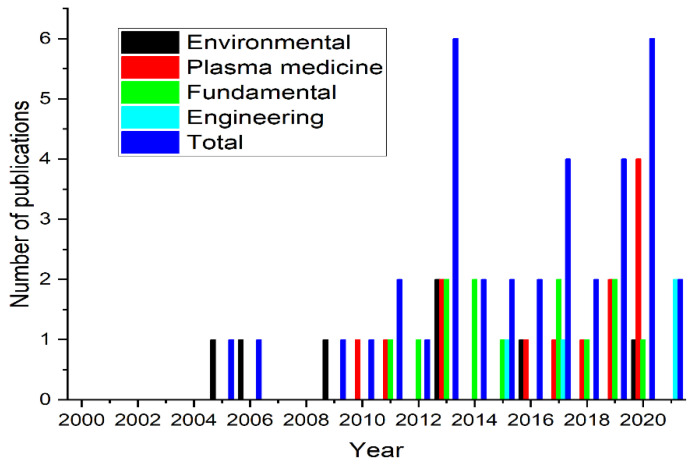
Distribution of published studies by year and macro research field.

Chronologically, research interests started with applications in the environmental area (2005–2009), followed by plasma medicine and the fundamental area in 2010 and 2011, respectively (Figure 3).

One of the main uses of plasma in the environmental sector is the purification of industrial wastewater, due to the production of ozone, which is a strong oxidant and can decompose toxic materials [61]. Thus, remediation by oxides of nitrogen (NO_x_) and sulfur (SO_2_), as well as the destruction of volatile organic components and heavy metals are common applications of plasma in this sector, which employs both highly energetic electron beams, corona discharges, and dielectric barrier discharges (DBDs), as well as other discharges in the treatment of solutions [62,63].

On the other hand, the remarkable potential of plasma in medical applications has led to the creation of a new and independent medical field called “plasma medicine” or “plasma biomedicine” [2]. With the growth of this area in the last 10 years, the need to investigate mechanisms and general reactions provoked by the treatment has emerged, which is directly related to the fundamental studies of plasma physics [2,64]. All figures and tables should be cited in the main text as Figure 1, Table 1, etc.

**Figure 4:**
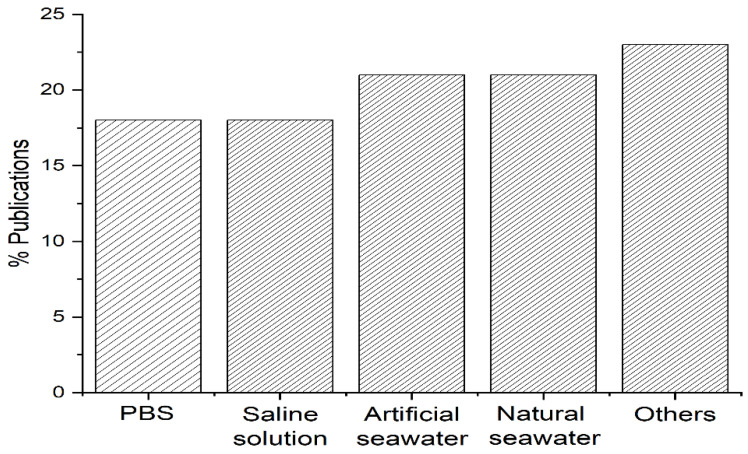
Types of plasma-treated saline water. PBS [22,39,40,44,51,57,60]; NaCl 0.9% [22,47,48,50,51,56,58]; artificial sea water [21,27,28,29,41,42,43,54]; natural sea water [19,20,23,24,25,26,38,59]; other saline solutions [30,31,32,45,46,49,52,53,55].

Depending on the purpose and, consequently, on the macro-research field in which the study is inserted, the types of treated saline waters may vary. In plasma medicine applications, for example, treatment with saline (0.9% NaCl) or phosphate-buffered saline (PBS) is common, particularly for disinfection purposes. In fact, two articles in the area comparatively used the treatment of both solutions, PBS and saline, in order to investigate the bactericidal effects and the reactive species generated by the plasma in the solutions [22,51]. In turn, fundamental, environmental, and engineering-related studies frequently report the treatment of seawater and other saline waters, representing a large part of the analytes identified in this review, as shown in Figure 4.

### 3.2. Plasma Sources

**Figure 5:**
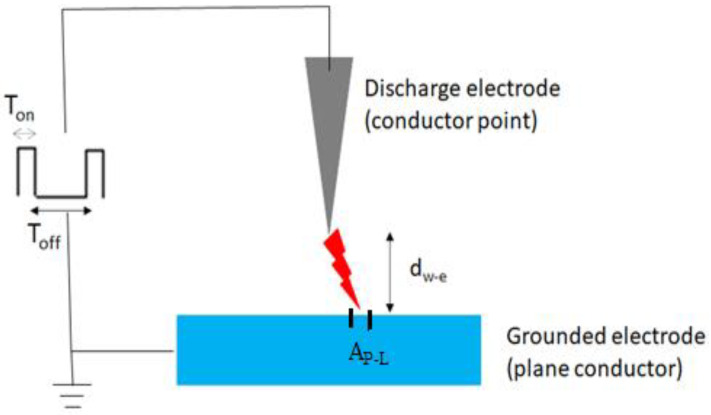
Schematic configuration of a corona discharge.

The plasma most used for the treatment of saline water has been the corona discharge configuration (35%). This configuration consists of applying a potential difference between a conducting point and a plane, to produce an electrical discharge with an effective area of plasma–liquid interaction (A_P-L_), of around 1 mm^2^ (Figure 5). In the present review, the conducting plane has always been saline water, with cathodic or anodic polarization and the discharges produced by pulsed voltages at a distance d_w-e_. Due to the short pulse duration (T_on_) and a sufficiently long pulse repetition time (T_off_) for electric arc feedback, all corona discharges in the present work can be classified as cold plasma [33].

**Figure 6:**
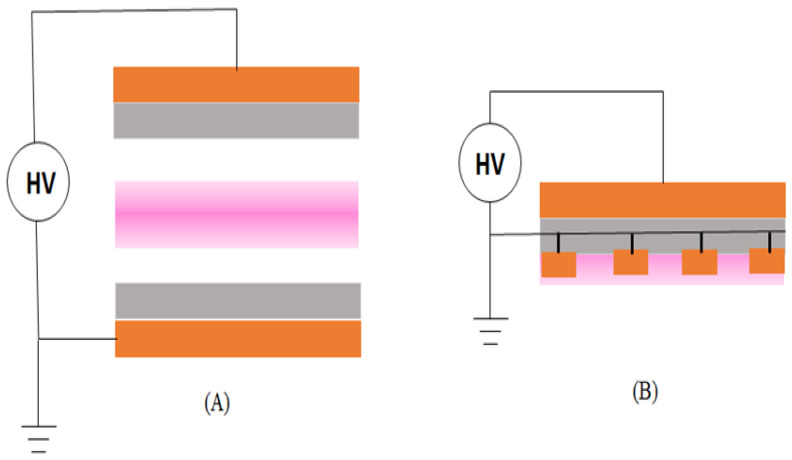
Dielectric barrier discharge configuration with (**A**) parallel and (**B**) coplanar electrodes.

The plasma generated in the DBD configuration represented 27% of the systems used in the literature. Generally, the electrical discharge is formed between two dielectric covered metal electrodes (Figure 6). In this case, the current is limited by the dielectric material, thus avoiding the thermal effect produced by electric arcs [65]. In general, the A_P–L_ in this configuration is higher than the corona discharge [66]. The DBD systems presented here are distinguished by electrode configuration (parallel, coplanar, cylindrical, porous electrodes), waveform (AC-sine, DC-square, DC-triangular, etc.), and/or frequency (from 50 Hz to 50 kHz) [66].

**Figure 7:**
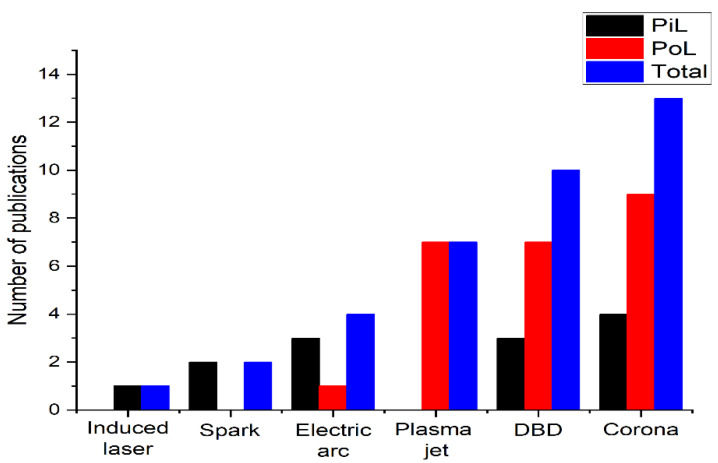
Plasma generation methods for saline-based water treatment, where PiL refers to the configuration in which the plasma is immersed in the liquid, and PoL to those configurations where the plasma is applied to the surface of the saline liquid.

We classified as plasma jet (19% of the total papers reviewed) any system that used inert gas or a gas mixture to transfer the plasma to the working or processing region [66]. This plasma can be produced by different power sources, but typically they are generated by DBD configuration, in the radiofrequency range [4]. Other types of discharge, employing micro arc and spark discharge, were reported in only 11% and 5% of the studies, respectively (Figure 7). One study also uses the method of producing plasma in saline water using laser [53].

In plasma jet treatments, most studies use He (99.996%) as carrier gas [44,46,47] or gas mixtures such as He + O_2_ (99.996% He + 0.3 to 0.5% O_2_) [39,51], as well as atmospheric air and N_2_ (99.9%) [22,60].

### 3.3. Plasma–Saline Water Interaction

#### 3.3.1. Chemical Reactions and Reactive Species

The plasma–saline water interaction follows the behavior pattern of the low conductivity plasma–liquid interaction, showing reductions in pH along the treatment time. Since changes in pH are directly related to the concentration of H^+^ ions in the solution, it means that the interaction between the chemical species of the plasma and the components of the solution triggers reactions that lead to an increase in [H^+^].

Acidification can also be altered with type of discharge polarization. When the discharge is anodic, there is a voltage drop on the surface of the solution, forming a cathodic sheath, which is responsible for the acceleration of ions from the plasma to the solution, inducing the emission of secondary electrons (e_2nd_^−^) or the decomposition of constituents of the liquid [67]. Some of these electrons move towards the plasma region to sustain the discharge, while others recombine in the liquid phase, taking the form of hydrated electrons (e_aq_^−^), which are strong reducing agents. On the other hand, when the discharge is the cathodic type, there is no high voltage drop at the liquid surface, so a flux of low-energy free electrons arrives in solution, producing reducing species such as e_aq_^−^, H, H_2_O_2_, and ultraviolet radiation. However, it has lower efficiency than anodic discharge due to the absence of energetic ions in the carrier gas, which trigger the formation of other radicals [67,68].

In the corona discharge configuration, the energy of the discharge is applied to a small A_P-L_ [66], producing localized acidification, which is then dispersed to uniform the solution [25]. In general, the reactions as the acidification of the solution, [H^+^], is proportional to the discharge voltage [69]. Table 2 illustrates this behavior; however, when the distance between the high voltage electrode and the surface of the solution is increased, the effect of increasing voltage on acidification can be reduced. This is observed in the second line of the table, for the case in which the authors carried out the treatment of the solution at distances of up to 15 mm.

When analyzing DBD systems, it is notable that acidification increases with the V/A_P-L_ ratio considered (Table 3). Comparing this ratio between voltage and discharge area (A_P-L_) of two solutions with the same NaCl concentration (9 g/L), we observe that the concentration of H^+^ protons is reduced from 5.0 × 10^−3^ to 1.7 × 10^−5^ mol/L when V/A_P-L_ reduces from 2241 V/cm^2^ to 1414 V/cm^2^ [48,56].

On the other hand, an inverse relationship with salinity was observed, i.e., the higher the saline concentration, the lower the [H^+^]. For PBS, the value of [H^+^] is practically constant, since it is a buffered solution [22,39,40,60]. Because of this, we estimated the amount of H^+^ ions produced by the plasma in the buffer through the Henderson–Hasselbalch equation (Equation (1)):

(1) $$\mathrm{pH}=\mathrm{pKa}+\log\frac{\left[A^{-}\right]}{\left[\mathrm{HA}\right]}$$

This equation allows the determination of pH as long as the concentration of acid and conjugate base is known, as well as the pKa. If a certain amount of acid is added to a buffered solution, some of the base in the buffer will be converted to conjugate acid, and the [A^−^]/[HA] ratio will change [70]. Knowing the pH variation, therefore, we can define the concentration of acid (H^+^) that was produced in the buffer to cause this variation. The calculation is done by estimating the acid value [HA] needed to vary the pH of a phosphate-buffered saline solution, whose pKa is 7.2. The values of this estimate are shown in Table 4.

Similarly, plasma-jet treated saline water demonstrated a reduction in pH as a function of increasing discharge voltage. In this configuration, considering the same discharge energy as in the previous configurations (corona and DBD), the energetic potential of the species is expected to be lower, considering that the plasma species are recombined during propagation through the carrier gas to the liquid surface. Consequently, the water–electrode distance (d_w-e_) and gas flow are important variables for the kinetics of the plasma–liquid reactions (Table 4).

By increasing d_w-e_ or decreasing the gas flow, the energy transferred to the plasma species at the surface of the solution will decrease. In other words, a change in this distance combined with variations in the gas flow rate can alter the nature of the discharge, modifying the distribution of excited species and the value of the electric field strength on ionization processes [47].

Acidification caused by plasma can occur with the injection of ions, which react with species in the saline water, such as salts and hydroxyl, forming H_3_O^+^ [71]. The mechanism of this process is mainly attributed to two pathways: (i) the interaction between electrons, H_2_O, OH^−^, and N_2_, resulting in the formation of acids such as HNO_3_ and HNO_2_ [19,21,49,56]; and (ii) the ionization of water by energetic particles of the plasma, which lead to an increase in the concentration of H^+^ in the solution, in a controlled atmosphere (absence of N_2_) [25]. As acidification occurs initially on the surface and then is homogenized in the volume of the solution, these changes may be more relevant for a higher V/A_P-L_ ratio [25].

Considering the first pathway for acidification reactions to occur, the existence of H^+^, NO_2_^−^, and NO_3_^−^ ions in the reaction medium is assumed. These ions are formed from reactive oxygen and nitrogen species (RONS) resulting from the ionization of atmospheric oxygen and nitrogen by the excited electrons of the plasma as a function of treatment time and precursor species of numerous products, such as NO, NO_2_, N_2_O_4_, and N_2_O_3_ [49,56]. Some of these products are responsible for the occurrence of other comparably important reactions in the plasma–saline water interaction, such as O_3_ and H_2_O_2_, responsible for the formation of OH, O_2_, HO_2_, HO_3_, hydroxynitrate (O_2_NOOH/O_2_NOO), and peroxynitrite (ONOOH/ONOO), with strong potential for oxidation and dissociation of other species [20,48]. These species are classified according to their stability as either short-lived or long-lived species. The short-lived species have a useful life of a few seconds, such as radicals, atomic oxygen, and peroxynitrite, while the long-lived species are considered biologically relevant due to their longer duration and range, such as nitrogen peroxide, nitrites, nitrates, etc. [72,73,74].

Parameters such as the nature and flow of gas, plasma–solution distance, and composition and volume of the liquid have an influence on the number of reactive species produced by the plasma. At distances of up to 30 mm from the surface, the main RONS formed is H_2_O_2_, whose production is favored by the increase in energy. At greater distances, the occurrence of reactions between the plasma species and the atmospheric air before reaching the interface reduces the concentration of H_2_O_2_ produced. Another factor directly related to the production of these species is the gas flow. By increasing the flow rate of a gas, less air is mixed into the channel, and the flow is greater, resulting in a decrease in gas-phase RONS. On the other hand, the increase in the volume of the liquid presents a behavior inversely proportional to the production of RONS; that is, decreases in the volume favor the increase of the concentration of RONS produced by the plasma in the medium [74].

In addition to the RONS production, reactive chlorine/oxy-chlorine species (RCS) are also generated by the plasma–saline water interaction. The production of these species grows with the increase of the energy of the species interacting with the surface of the solution, effective area of interaction, and treatment time. In saline water, the chlorine radical can reduce the concentration of NO_2_^−^ and increase the pH of the reaction medium due to the occurrence of Equation (2) [21,39,48,51] as follows:

HClO + NO_2_^−^ → ClNO_2_ + OH^−^ (2)

On the other hand, the presence of Cl^−^ ions can favor an increase in the concentration of H^+^ with the interaction by the plasma, since, like other anions of halides, it can combine with OH radicals from Equations (3)–(6) [75]:

•OH + Cl^−^ → Cl• + OH^−^ (3)

Cl• + Cl• → Cl_2_ (4)

Cl_2_ + H_2_O → HCl + HClO (5)

2HClO + H_2_O_2_ → 2Cl− + 1O_2_ + 2H^+^ (6)

Allied to this, the use of high voltages in atmospheric plasmas tends to favor the occurrence of these and other reactions, considering that more energy is supplied in a single pulse, producing a greater number of reactive species as a consequence of the abundance of energetic electrons in the medium [76,77]. These energetic electrons and positive ions participate in important equations, such as the following [1,78]:

H_2_O + e → H_2_O^+^ + 2e (7)

H_2_O^+^ + H_2_O → H_3_O^+^ + OH* (8)

M^+^ + H_2_O → M + H_2_O^+^ (9)

H_2_O^+^ + H_2_O → H_3_O^+^ + OH (10)

An increase in voltage from 16 kV to 21 kV for corona discharge in seawater, for example, can increase the energy efficiency rate due to the higher electric field strength in these circumstances [27].

The detection of reactive species from the plasma in saline water can be done immediately during the treatment using the Optical Emission Spectrometry Technique (OES), which allows for the identification of the spectra of the species produced in the reaction medium, as well as using comparative methods of chemical analysis of saline water before and after treatment. Of the included studies, 37% of the total used OES to determine the RONS, while 27% used other methods such as spectroscopy and titration to determine the presence of these species; the rest of the studies do not mention the method used for this purpose.

#### 3.3.2. Plasma-Induced Salt Crystallization

Since the ionic composition of saline water is modified by the interaction with the plasma, there is an increase in conductivity as a result of the accumulation of reactive species in the liquid [42,50,79]. However, this effect is limited by plasma-induced salt crystallization and solution evaporation, which reduce the amount of dissociated ions [26].

One of the consequences of the increase in conductivity caused by the plasma is the greater formation and emission of some active aqueous species, such as the •OH e H_2_O_2_ [39,47,51]. However, the initial conductivity of the solution is a factor of similar importance for the species formation process, in different gas discharge configurations.

For example, in a low conductivity solution subjected to plasma jet treatment, the discharge behaves like streamers, producing a high electron density, whereas in a high conductivity solution, a bubble plasma with low electron density is generally formed, which is influenced by variation in the gas flow rate, mainly around 1000 µS/cm [80].

For pulsed corona discharges, the low conductivity of the liquid (less than 10 μS/cm) results in a narrow voltage range capable of producing a corona discharge with no sparking. Above 400 μS/cm, the streamers become short, and the efficiency of radical production decreases, generating plasma with reduced gas temperature. In short, the production of radicals, such as OH and atomic oxygen, in this case, is more efficient at conductivities lower than 100 μS/cm [78].

Overall, for cold plasma, changes in conductivity are related to instabilities at the plasma–saline interface. When the conductivity of the liquid is greater than the conductivity of the adjacent plasma, the surface is not easily deformed with treatment. However, if the conductivity of the liquid is less than that of the plasma, the current density distribution is unstable, and deformations at the interface can be formed as a function of the applied current density. If the density is low, instabilities can be eliminated by the surface tension of the liquid, but at higher current densities, evaporation can cause voltage drops at the interface and the formation of streamers within the liquid volume [45].

It is known that the stability limit of a planar water–air interface tensioned by a homogeneous and perpendicular electric wire is given by Equation (11) [69]:

(11) $$E=\sqrt{\frac{2\sigma }{\epsilon }\sqrt{\frac{\rho g}{\gamma }}}$$

Thus, liquid surface instabilities occur when the electric field strength (E) becomes stronger than the gravitational force (g), and the surface tension force (γ) can compensate. That is, an increase in electrical force near the surface, caused by an increase in voltage, causes instabilities, the shape of which is called a Taylor cone [69]. These instabilities occur due to the reordering of the dipoles towards the discharge, when the electric field is applied [81].

In saline water (electrolytes), the surface is deformed differently depending on the density of dissolved ions, even under the action of a constant electric field, as these species can change the surface tension of the liquid. Furthermore, the instabilities also vary as a function of the distance between the electrode and the surface of the solution (d_w-e_). Decreasing d_w-e_ can mean lower breakdown voltage and higher discharge current, increasing surface deformation as a result of greater dissolution of ions in the electrolyte [82].

In solutions with a high concentration of salts, such as seawater and brines, surface instabilities caused by electrical discharge can be accompanied by the formation of crystallized salts. This event occurs immediately after the breakdown between the electrode and the surface of the solution, forming a layer of salts that can reach the electrode (saline bridge) due to the weakening of the solvation layer of the ions by the applied electric field [26].

Another important fact is that changes in gas discharge polarization can trigger different crystallization mechanisms. A cathodic discharge is able to form a Taylor cone on the surface and provide crystallization, because the polarity of the plasma attracts the salt-forming cations to the outermost layer of the liquid. On the other hand, in anodic discharge, the surface layer of the liquid is mainly composed of anions and, since it is bombarded with positive ions, the salt particles are not created or ejected towards the electrode, tending to remain in solution [25]. Furthermore, the anodic configuration causes a pronounced additional effect of evaporation of the saline water by localized heating, as observed by Alves-Junior et al. [25].

#### 3.3.3. Evaporation

The evaporation associated with heating of the solution is a factor that favors crystallization. In the evaporation of saline water, only the water evaporates, and the salts remain in solution, leading to a decrease in partial vapor pressure as a function of heating time, until the liquid approaches the point of crystallization [83]. Due to the change in equilibrium caused by the increase in the concentration of salts, greater heating is necessary to maintain the evaporation rate of the saline water, which precedes crystallization [83]. Studies reporting the effect of plasma-induced evaporation in saline solutions included here mainly used the CAP in the corona discharge configuration (Table 5).

It was observed that this effect can be more pronounced in the anodic configuration of corona discharge due to the greater localized heating effect mentioned earlier. In addition, when applied in saline water, this type of electrical discharge caused an increase in the temperature of the liquid of 75 °C in 10 min of interaction, causing evaporation [19].

However, the increase in evaporation rate with exposure to plasma is not necessarily limited by temperature. In circumstances where plasma treatment took place at temperatures below the boiling point, the estimated evaporation rate was similar to the case of treatments in the 3000 K range [84]. In Maguire et al., the diameter of a droplet exposed to atmospheric plasma decreased from 15 μm to 13 μm under conditions in which the gas temperature was lower than 400 K, which indicates that the droplet size reduction may have been influenced by factors beyond standard heating evaporation, such as chemical effects of loading and the surface [84].

Interaction with the plasma sheath and evaporation of volatile species by electron bombardment are possible ways to explain this behavior. Formation of volatile species on the surface due to electron bombardment can lead to increased evaporation, while microdroplet loading and impact on plasma conditions are also important factors [84]. It was observed that the plasma sheath increased with the introduction of water droplets and, considering that the evaporation rate measured for the case of varying temperatures was similar, the fragmentation of the droplet surface due to charge-induced instabilities may also have an impact on plasma and fragment heating [84].

Another effect to consider is the gas flow rate used in the treatment. In plasma jet using helium gas, it has been shown that saline water evaporation can be induced by flow, which governs the thermal effect originating from liquid cooling. Considering that in the plasma jet configuration studied the only possible sources of thermal effects are heating due to plasma discharge and liquid cooling due to evaporation caused by helium flow (low moisture content), it was found that the average surface temperature variation shows a predominant cooling effect, being essentially affected by gas flow. This assumption is confirmed considering that the gas temperature at the capillary outlet was recorded as between 21 and 22 °C and increasing to 24.6 °C in the presence of plasma; however, upon reaching the solution, the gas is cooled by the evaporation of water and reaches temperatures of 17–19 °C, which suggests that any possible thermal effect is controlled by gas-induced evaporation [85].

Contrary to what happens when a saline water is exposed to natural evaporation, the salts crystallized under the influence of plasma tend to have a characteristic morphology, with a smaller size, in addition to uneven precipitation, as a result of the greater reaction kinetics provided by the plasma [26]. As expected, a change in gas discharge polarization also has different effects on this process. In seawater subjected to a dielectric barrier discharge, for example, a reduction in the size of the salt particles from 9.98 μm to 0.58 μm in positive discharge and from 9.98 μm to 0.41 μm in negative discharge was observed by Kim et al. [23]. These effects are at an early stage of investigation and require further study.

## 4. Conclusions

The plasma–saline water interaction has emerged as a field of study since 2005 and has important applicability in several areas, of which fundamental studies and plasma medicine stand out, with a view to modifying the properties of saline waters and physiological solutions, mainly. These solutions have been commonly treated with cold atmospheric plasmas, especially in corona and DBD configurations, in PoL systems.

The ionic concentration of the liquid and the polarization of the gas discharge provide changes in the properties of the solution, such as an increase in the concentration of reactive species and conductivity; pH reduction as a function of voltage increase, effective interaction area (A_P-L_), and discharge–solution distance (d_w-e_); as well as deformations on the surface and localized evaporation, accompanied by the crystallization of dissolved salts. The crystals formed under these conditions have a characteristic morphology as a result of the higher reaction kinetics caused by the discharge.

In view of this, the interaction between cold atmospheric plasma and this type of solution appears to be a promising field for the development of new technologies, whether in desalination and salt extraction or in improving the properties of the liquid. Depending on the purpose of activating a saline plasma solution, one configuration may be more favorable than another. The corona discharge, for example, is more localized, since the electrical discharge mostly occurs in a needle electrode. Consequently, localized heating can occur, which makes it a tool with related applications for evaporation of the liquid and precipitation and crystallization of salts. DBD and plasma jet, on the other hand, can be interesting when this localized heating is not desired, as in the treatment of biological samples, making it possible to modify the properties of the liquid by producing active radicals at variable surface sizes.

### Summary Definitions of the Solutions and Main Effects of Plasma

**Natural sea water:** water obtained directly from the sea with an estimated saline concentration of 37.25 g/L of dissolved salts, most of the time. Some studies reported here use this evaporated water, which leads to an increase in the concentration of salts in a liquid medium. The main reactions triggered by the plasma in this type of solution are: solution evaporation; induction of salt precipitation and consequent reduction of the conductivity of the solution by extraction of these salts; drop in pH due to the formation of HNO_2_ and HNO_3_, as well as autoionization of H_2_O by plasma ions.

**Artificial sea water:** saline water produced in the laboratory, simulating the composition of natural sea water, with a salinity of approximately 37.25 g/L of dissolved salts, of which 26.75 are NaCl; 4.88 MgCl_2_; 3.54 MgSO_4_; 0.72 KCl; 1.16 CaCl_2_; and 0.2 NaHCO_3_. When plasma treated, this type of solution has effects similar to those of natural seawater.

**0.9% NaCl saline solution:** Also known as saline solution, it is a solution prepared with a concentration of 9 g/L of NaCl, widely used in sterilization and disinfection processes. The main effects of plasma treatment in this type of solution are: reduction in pH and increase in conductivity due to the production of RONS, such as NO_2_, NO_3_, O_3_, OH, and H_2_O_2_, also responsible for potentiating the bactericidal effect of this solution.

**Phosphate-Buffered Saline (PBS):** Saline with a concentration of approximately 9.86 g/L of dissolved salts, of which 8.00 are NaCl; 0.20 is KCl; 1.42 is Na_2_HPO_4_; and 0.24 is KH_2_PO_4_. Commonly used in biochemistry, it is an isotonic solution that is non-toxic to human body cells. When treated by plasma, this solution, contrary to what happens in other solutions, does not present significant pH variations due to the buffering of the solution; however, the plasma favors the increase of the concentration of RONS, causing a bactericidal effect in the solution.

## Figures and Tables

**Table 1:** List of articles included in the review.

Reference	Year	Main Objective
[26]	2021	To explore the crystallization mechanisms on the surface of hypersaline droplets with and without application of corona discharge.
[25]	2021	To investigate the influence of cathodic and anodic pulsed corona discharge in hypersaline water.
[30]	2016	Trace mechanisms for obtaining dye transformation products in non-thermal plasma-treated brines.
[20]	2005	To investigate the effect of non-thermal plasma in the treatment of red tide in sea water.
[38]	2010	To present the effects of the hydroxyl radical on algae and bacteria in plasma-treated seawater.
[22]	2013	To investigate the bactericidal effects of five plasma-treated saline solutions.
[24]	2017	To understand the mechanism of crystallization in mother liquor caused by the application of plasma.
[39]	2020	Systematically analyze reactive species generated by helium plasma jet interaction with saline solutions.
[40]	2011	To inactivate *E. coli* using plasma-treated water + PBS solution.
[19]	2020	To use atmospheric plasma for desalination of seawater and crystallization of its salts.
[41]	2019	To investigate effects of micro-arc discharges in highly conductive liquids.
[42]	2017	Studying the needle-plane micro-plasma system in seawater.
[43]	2018	To examine micro-plasma generation by micro-arc discharges in seawater.
[44]	2020	To monitor the helium plasma jet in water + PBS solution and analyze current parameters and excited species.
[28]	2013	To catalyze oxidation of S(IV) to S(VI) by high voltage discharge in artificial seawater.
[45]	2015	To study changes in the plasma–salt liquid interface in a small surface area.
[46]	2019	To evidence the influence of the plasma–target distance on the electric field profile.
[32]	2020	To assess the potential of plasma-activated brine as a source of nitrite.
[23]	2013	To investigate the performance of capillary membrane discharge on the inactivation of microorganisms in seawater.
[47]	2017	To study the effects of the collision of the plasma jet with the surface of the conducting liquid.
[48]	2016	Numerically study the interaction between a surface discharge and a downstream discharge in saline.
[49]	2019	To investigate effects of plasma-treated brine on meat curing.
[50]	2013	To evaluate physicochemical properties of deionized water and saline treated with plasma.
[51]	2018	To investigate the potential for penetration into saline water of nitrogen species generated by plasma.
[52]	2014	To investigate the generation and propagation of shock waves in saline water.
[21]	2011	Examine characteristics and possibility of inactivation of *E. coli* from plasma-treated seawater.
[53]	2015	To study the influence of salinity in aqueous media in the laser-induced plasma machining process.
[27]	2009	To show a novel process of oxidation from S(IV) to S(VI) by pulsed corona discharge in seawater.
[54]	2012	To study effects of multichannel underwater electrical discharge in saline water.
[55]	2020	To evaluate the physicochemical properties and bactericidal activities of plasma-activated saline water.
[56]	2019	To investigate processes and mechanisms of sterilization of plasma in saline solution.
[29]	2013	To investigate the behavior of corona discharge in saline water in S(IV) oxidation.
[57]	2006	Examine the fundamental characteristics of a PBS arc discharge system.
[31]	2017	To evaluate the potential of plasma-treated water as an alternative to synthetic sodium nitrite.
[58]	2013	To investigate the influence of voltage pulse frequency on plasma bubble production in saline solution.
[59]	2014	To investigate the mechanism of bubble generation by underwater discharge in saline solution.
[60]	2020	To investigate the effectiveness of inactivation of microorganisms of plasma treatment in five liquid media.

**Table 2:** Acidification of saline water by corona discharge.

Solution	Composition ^1^ (%)	Saline Concentration (g/L)	Process Parameters	Treatment Time (min.)	∆[H^+^] (mol/L)	Ref.
Natural sea water	NaCl 72.2 MgCl_2_ 13.2 MgSO_4_ 9.5 CaCl_2_ 3.1 KCl 1.9	37.05	10 kV 20 kHz	15	7.59 × 10^−3^	[19]
Artificial sea water	NaCl 71.8 MgCl_2_ 13.1 MgSO_4_ 9.5 CaCl_2_ 3.1 KCl 1.9 NaHCO_3_ 0.5	37.25	70 kV -	-	2.50 × 10^−3^	[29]
Artificial sea water	NaCl 72.2 MgCl_2_ 13.2 MgSO_4_ 9.5 CaCl_2_ 3.1 KCl 1.9	37.05	5 kV 10 kHz	5	2.58 × 10^−9^	[21]
Physiological serum	NaCl 0.9	9.00	0.6 kV 2.8 × 10^8^ kHz	40	−2.36 × 10^−7^	[50]

^1^ Some values for sea water are estimates.

**Table 3:** Acidification of saline water by DBD.

Solution	Composition (%)	Saline Concentration (g/L)	Parameters	V/A_P-L_ (V/cm²)	Vol. (mL)	Time (min.)	∆[H^+^] ^2^ (mol/L)	Ref.
PBS	NaCl 81.1 KCl 2.0 Na_2_HPO_4_ 14.4 KH_2_PO_4_ 2.4	9.86	20 kV 7 kHz	3945	2	2	9.36 × 10^−3^	[40]
Physiological serum	NaCl 100	9.00	44 kV 0.6 kHz	2241	2	1–5	5.01 × 10^−3^	[56]
Saline solution 1	NaCl 97.5 KCl 2.4	8.20	80 kV 0.05 kHz	1258	10	10	9.80 × 10^−4^	[55]
Physiological serum	NaCl 100	9.00	10 kV 50 kHz	1414	7	1.5	1.69 × 10^−5^	[48]
Saline solution 2	Na_4_P_2_O_7_ 100	5.00	70 kV 50 kHz	396	785	1–6	9.97 × 10^−7^	[49]
Natural sea water	NaCl 72.2 MgCl_2_ 13.2 MgSO_4_ 9.5 CaCl_2_ 3.1 KCl 1.9	37.05	2 kV 10 kHz	3.97	4032	-	1.73 × 10^−9^	[20]

^2^ [H^+^] produced by plasma in buffered saline solutions was estimated from the Henderson–Hasselbalch equation.

**Table 4:** Acidification of saline water by atmospheric plasma jet.

Solution	Composition (%)	Saline Concentration (g/L)	Parameters	d_w-e_ (mm)	Time (min.)	∆[H^+^] ^3^ (mol/L)	Ref.
PBS	NaCl 81.1 KCl 2.0 Na_2_HPO_4_ 14.4 KH_2_PO_4_ 2.4	9.86	30 kV -	80	5	4.95 × 10^−3^	[60]
PBS	NaCl 81.1 KCl 2.0 Na_2_HPO_4_ 14.4 KH_2_PO_4_ 2.4	9.86	16 kV 8 kHz	5	5–20	3.80 × 10^−3^	[51]
Physiological serum	NaCl 100	9.00	1 kV 10 kHz	2	20	3.15 × 10^−4^	[22]
Physiological serum	NaCl 100	9.00	16 kV 8 kHz	5	5–20	2.16 × 10^−7^	[51]

^3^ [H^+^] for PBS are estimate by Equation (1).

**Table 5:** Plasma-induced evaporation in saline water.

Solution	Plasma Source and Device	Parameters	Treatment Time (min.)	Initial Volume (mL)	Final Volume (mL)	Ref.
Natural sea water	CAP—Corona discharge	10 kV 20 kHz	15	40.00	25.00	[19]
Evaporated natural sea water	CAP—Corona discharge	2.3 kV -	30	4.20	3.76 (anodic) 4.15 (cathodic)	[25]
Evaporated natural sea water	CAP—Corona discharge	11 kV 500 Hz	03	0.05	0.00	[26]

## Data Availability

Not applicable.

## References

[1] Yang Y., Cho Y.I., Fridman A. (2012). Plasma Discharge in Liquid.

[2] Rezaei F., Vanraes P., Nikiforov A., Morent R., De Geyter N. (2019). Applications of Plasma-Liquid Systems: A Review. Materials.

[3] Mariotti D., Patel J., Švrček V., Maguire P. (2012). Plasma-liquid interactions at atmospheric pressure for nanomaterials synthesis and surface engineering. Plasma Process. Polym..

[4] Surowsky B., Schlüter O., Knorr D. (2015). Interactions of Non-Thermal Atmospheric Pressure Plasma with Solid and Liquid Food Systems: A Review. Food Eng. Rev..

[5] Vandenabeele C.R., Lucas S. (2020). Technological challenges and progress in nanomaterials plasma surface modification—A review. Mater. Sci. Eng. R Rep..

[6] Huang Y., Yu Q., Li M., Sun S., Zhao H., Jin S., Fan J., Wang J. (2021). An overview of low-temperature plasma surface modification of carbon materials for removal of pollutants from liquid and gas phases. Plasma Process. Polym..

[7] Fiebrandt M., Lackmann J.-W., Stapelmann K. (2018). From patent to product? 50 years of low-pressure plasma sterilization. Plasma Process. Polym..

[8] Puač N., Gherardi M., Shiratani M. (2018). Plasma agriculture: A rapidly emerging field. Plasma Process. Polym..

[9] Sakudo A., Yagyu Y. (2021). Plasma Biology. Int. J. Mol. Sci..

[10] Bekeschus S., Favia P., Robert E., von Woedtke T. (2019). White paper on plasma for medicine and hygiene: Future in plasma health sciences. Plasma Process. Polym..

[11] Yan X., Ouyang J., Zhang C., Shi Z., Wang B., Ostrikov K. (2019). Plasma medicine for neuroscience—An introduction. Chin. Neurosurg. J..

[12] Liu D., Szili E.J., Ostrikov K. (2020). Plasma medicine: Opportunities for nanotechnology in a digital age. Plasma Process. Polym..

[13] Tabares F.L., Junkar I. (2021). Cold Plasma Systems and Their Application in Surface Treatments for Medicine. Molecules.

[14] Misra N.N., Yepez X., Xu L., Keener K. (2019). In-package cold plasma technologies. J. Food Eng..

[15] Perinban S., Orsat V., Raghavan V. (2019). Nonthermal Plasma–Liquid Interactions in Food Processing: A Review. Compr. Rev. Food Sci. Food Saf..

[16] Waghmare R. (2021). Cold plasma technology for fruit based beverages: A review. Trends Food Sci. Technol..

[17] Locke B.R., Sato M., Sunka P., Hoffmann M.R., Chang J.S. (2006). Electrohydraulic discharge and nonthermal plasma for water treatment. Ind. Eng. Chem. Res..

[18] Bruggeman P., Leys C. (2009). Non-thermal plasmas in and in contact with liquids. J. Phys. D Appl. Phys..

[19] Ekanayake U.G.M., Seo D.H., Faershteyn K., O’Mullane A.P., Shon H., MacLeod J., Golberg D., Ostrikov K. (2020). Atmospheric-pressure plasma seawater desalination: Clean energy, agriculture, and resource recovery nexus for a blue planet. Sustain. Mater. Technol..

[20] Bai M., Bai X., Zhang Z., Bai M., Yang B. (2005). Treatment of red tide in ocean using non-thermal plasma based advanced oxidation technology. Plasma Chem. Plasma Process..

[21] Ryu S.M., Hong E.J., Seok D.C., Yoo S.R., Kim Y.J., Lho T., Lee B.J. (2011). Characteristics of discharged sea water generated by underwater plasma system. Curr. Appl. Phys..

[22] Baik K.Y., Kim Y.H., Ryu Y.H., Kwon H.S., Park G., Uhm H.S., Choi E.H. (2013). Feeding-gas effects of plasma jets on *Escherichia coli* in physiological solutions. Plasma Process. Polym..

[23] Kim Y.J., Hong Y.C., Lee S.J., Kim J.H., Lee B.J. (2013). Underwater capillary discharge on the penetrability of a membrane. Surf. Coat. Technol..

[24] de Barauna J.B.F.O., Pereira C.S., Gonçalves I.A., De Oliveira Vitoriano J., Junior C.A. (2017). Sodium chloride crystallization by electric discharge in brine. Mater. Res..

[25] Alves-Junior C., Rodrigues-Junior F.E., Vitoriano J.O., Barauna J.B.F.O. (2021). Investigating the Influence of the Pulsed Corona Discharge Over Hypersaline Water. Mater. Res..

[26] Almada L.F.A., Fontes K.E.S., Vitoriano J.O., Melo V.R.M., Fraga F.E.N., Alves C. (2020). Applying pulsed corona discharge in hypersaline droplets. J. Phys. D Appl. Phys..

[27] Shi N., Zhang X., Lei L. (2009). Sulfite oxidation in seawater flue gas desulfurization by a pulsed corona discharge process. Sep. Purif. Technol..

[28] Gong J., Zhang X., Wang X., Lei L. (2013). Oxidation of S(IV) in seawater by pulsed high voltage discharge plasma with TiO_2_/Ti electrode as catalyst. Plasma Sci. Technol..

[29] Wang X., Li Z., Lan T., Lei L. (2013). Sulfite oxidation in seawater flue gas desulfurization by plate falling film corona-streamer discharge. Chem. Eng. J..

[30] Azerrad S.P., Lütke Eversloh C., Gilboa M., Schulz M., Ternes T., Dosoretz C.G. (2016). Identification of transformation products during advanced oxidation of diatrizoate: Effect of water matrix and oxidation process. Water Res..

[31] Yong H.I., Park J., Kim H.-J., Jung S., Park S., Lee H.J., Choe W., Jo C. (2017). An innovative curing process with plasma-treated water for production of loin ham and for its quality and safety. Plasma Process. Polym..

[32] Inguglia E.S., Oliveira M., Burgess C.M., Kerry J.P., Tiwari B.K. (2020). Plasma-activated water as an alternative nitrite source for the curing of beef jerky: Influence on quality and inactivation of Listeria innocua. Innov. Food Sci. Emerg. Technol..

[33] Tendero C., Tixier C., Tristant P., Desmaison J., Leprince P. (2006). Atmospheric pressure plasmas: A review. Spectrochim. Acta Part B At. Spectrosc..

[34] Fricke K., Koban I., Tresp H., Jablonowski L., Schröder K., Kramer A., Weltmann K.D., von Woedtke T., Kocher T. (2012). Atmospheric pressure plasma: A high-performance tool for the efficient removal of biofilms. PLoS ONE.

[35] Park G.Y., Park S.J., Choi M.Y., Koo I.G., Byun J.H., Hong J.W., Sim J.Y., Collins G.J., Lee J.K. (2012). Atmospheric-pressure plasma sources for biomedical applications. Plasma Sources Sci. Technol..

[36] Stryczewska H.D., Jakubowski T., Kalisiak S., Gizewski T., Pawlat J. (2013). Power systems of plasma reactors for non-thermal plasma generation. J. Adv. Oxid. Technol..

[37] Page M.J., McKenzie J.E., Bossuyt P.M., Boutron I., Hoffmann T.C., Mulrow C.D., Shamseer L., Tetzlaff J.M., Akl E.A., Brennan S.E. (2021). The PRISMA 2020 statement: An updated guideline for reporting systematic reviews. Syst. Rev..

[38] Bai M., Zhang Z., Xue X., Yang X., Hua L., Fan D. (2010). Killing effects of hydroxyl radical on algae and bacteria in ship’s ballast water and on their cell morphology. Plasma Chem. Plasma Process..

[39] Chen T.-W., Liu C.-T., Chen C.-Y., Wu M.-C., Chien P.-C., Cheng Y.-C., Wu J.-S. (2020). Analysis of Hydroxyl Radical and Hydrogen Peroxide Generated in Helium-Based Atmospheric-Pressure Plasma Jet and in Different Solutions Treated by Plasma for Bioapplications. ECS J. Solid State Sci. Technol..

[40] Coleman J., Yost A., Goren R., Fridman G., Lowman A. (2011). Nonthermal atmospheric pressure plasma decontamination of protein-loaded biodegradable nanoparticles for nervous tissue repair. Plasma Med..

[41] Gamaleev V., Furuta H., Hatta A. (2019). Atomic Emission Spectroscopy of Microarc Discharge in Sea Water for On-Site Detection of Metals. IEEE Trans. Plasma Sci..

[42] Gamaleev V., Oh J.S., Furuta H., Hatta A. (2017). Investigation of effect of needle electrode configuration on microplasma discharge process in sea water. IEEE Trans. Plasma Sci..

[43] Gamaleev V., Furuta H., Hatta A. (2018). Generation of micro-arc discharge plasma in highly pressurized seawater. Appl. Phys. Lett..

[44] Gerber I.C., Mihaila I., Pohoata V., Topala I. (2020). Evolution of Electrical and Optical Parameters of a Helium Plasma Jet in Interaction with Liquids. IEEE Trans. Plasma Sci..

[45] Hoffer P., Kolacek K., Stelmashuk V., Lukes P. (2015). Penetration of Gas Discharge Through the Gas-Liquid Interface into the Bulk Volume of Conductive Aqueous Solution. IEEE Trans. Plasma Sci..

[46] Hofmans M., Sobota A. (2019). Influence of a target on the electric field profile in a kHz atmospheric pressure plasma jet with the full calculation of the Stark shifts. J. Appl. Phys..

[47] Kovačević V.V., Sretenović G.B., Slikboer E., Guaitella O., Sobota A., Kuraica M.M. (2017). The effect of liquid target on a nonthermal plasma jet—imaging, electric fields, visualization of gas flow and optical emission spectroscopy. J. Phys. D Appl. Phys..

[48] Liu Z.C., Guo L., Liu D.X., Rong M.Z., Chen H.L., Kong M.G. (2016). Chemical Kinetics and Reactive Species in Normal Saline Activated by a Surface Air Discharge. Plasma Process. Polym..

[49] Luo J., Yan W., Nasiru M.M., Zhuang H., Zhou G., Zhang J. (2019). Evaluation of physicochemical properties and volatile compounds of Chinese dried pork loin curing with plasma-treated water brine. Sci. Rep..

[50] Mystkowska J., Dabrowski J.R., Kowal K., Niemirowicz K., Car H. (2013). Physical and chemical properties of deionized water and saline treated with low-pressure and low-temperature plasma. Chemik.

[51] Nie L., Yang Y., Duan J., Sun F., Lu X., He G. (2018). Effect of tissue thickness and liquid composition on the penetration of long-lifetime reactive oxygen and nitrogen species (RONS) generated by a plasma jet. J. Phys. D Appl. Phys..

[52] Oshita D., Hosseini S.H.R., Mawatari K., Nejad S.M., Akiyama H. (2014). Two Successive Shock Waves Generated by Underwater Pulse Electric Discharge for Medical Applications. IEEE Trans. Plasma Sci..

[53] Saxena I., Ehmann K., Cao J. (2015). High throughput microfabrication using laser induced plasma in saline aqueous medium. J. Mater. Process. Technol..

[54] Stelmashuk V., Hoffer P. (2012). Shock Waves Generated by an Electrical Discharge on Composite Electrode Immersed in Water With Different Conductivities. IEEE Trans Plasma Sci.

[55] Tsoukou E., Bourke P., Boehm D. (2020). Temperature Stability and Effectiveness of Plasma-Activated Liquids over an 18 Months Period. Water.

[56] Wang H., Zhang L., Luo H., Wang X., Tie J., Ren Z. (2019). Sterilizing Processes and Mechanisms for Treatment of *Escherichia coli* with Dielectric-Barrier Discharge Plasma. Appl. Environ. Microbiol..

[57] Yamatake A., Angeloni D.M., Dickson S.E., Emelko M.B., Yasuoka K., Chang J.-S. (2006). Characteristics of Pulsed Arc Electrohydraulic Discharge for Eccentric Electrode Cylindrical Reactor using Phosphate-Buffered Saline Water. Jpn. J. Appl. Phys..

[58] Yoon S.-Y., Kim S.-J., Lee S.-H., Hong J.W., Kim K.-H., Seol Y.-J., Kim G.-H. (2013). Driving frequency dependency of gas species in the bubble formation for aqua-plasma generation. Curr. Appl. Phys..

[59] Zhao P., Roy S. (2014). A modified resistance equation for modeling underwater spark discharge with salinity and high pressure conditions. J. Appl. Phys..

[60] Zhao Y., Ojha S., Burgess C.M., Sun D., Tiwari B.K. (2020). Influence of various fish constituents on inactivation efficacy of plasma-activated water. Int. J. Food Sci. Technol..

[61] Xu X. (2001). Dielectric barrier discharge properties and applications. Thin Solid Film..

[62] Penetrante B.M., Schultheis S.E. (1993). Non-Thermal Plasma Techniques for Pollution Control.

[63] Becker K., Koutsospyros A., Yin S.M., Christodoulatos C., Abramzon N., Joaquin J.C., Brelles-Mariño G. (2005). Environmental and biological applications of microplasmas. Plasma Phys. Control Fusion.

[64] Weltmann K.-D., von Woedtke T. (2017). Plasma medicine—current state of research and medical application. Plasma Phys. Control Fusion.

[65] Subedi D.P., Joshi U.M., Wong C.S. (2017). Plasma Science and Technology for Emerging Economies: An AAAPT Experience.

[66] Ehlbeck J., Schnabel U., Polak M., Winter J., von Woedtke T., Brandenburg R., von dem Hagen T., Weltmann K.-D. (2011). Low temperature atmospheric pressure plasma sources for microbial decontamination. J. Phys. D Appl. Phys..

[67] Chen Q., Li J., Li Y. (2015). A review of plasma–liquid interactions for nanomaterial synthesis. J. Phys. D Appl. Phys..

[68] Chen Q., Kaneko T., Hatakeyama R. (2012). Reductants in Gold Nanoparticle Synthesis Using Gas–Liquid Interfacial Discharge Plasmas. Appl. Phys. Express.

[69] Bruggeman P., Graham L., Degroote J., Vierendeels J., Leys C. (2007). Water surface deformation in strong electrical fields and its influence on electrical breakdown in a metal pin-water electrode system. J. Phys. D Appl. Phys..

[70] Harris D.C., Daniel C. (2009). Harris—Explorando a Química Analítica.

[71] Chen Q., Saito K., Takemura Y.I., Shirai H. (2008). Physicochemistry of the plasma-electrolyte solution interface. Thin Solid Film..

[72] Girard P.-M., Arbabian A., Fleury M., Bauville G., Puech V., Dutreix M., Sousa J.S. (2016). Synergistic Effect of H_2_O_2_ and NO_2_ in Cell Death Induced by Cold Atmospheric He Plasma. Sci. Rep..

[73] Mohades S., Laroussi M., Sears J., Barekzi N., Razavi H. (2015). Evaluation of the effects of a plasma activated medium on cancer cells. Phys. Plasmas.

[74] Khlyustova A., Labay C., Machala Z., Ginebra M.-P., Canal C. (2019). Important parameters in plasma jets for the production of RONS in liquids for plasma medicine: A brief review. Front. Chem Sci. Eng..

[75] Wang X., Zhou M., Jin X. (2012). Application of glow discharge plasma for wastewater treatment. Electrochim. Acta.

[76] Wang Z., Jiang S., Liu K. (2014). Treatment of wastewater with high conductivity by pulsed discharge plasma. Plasma Sci. Technol..

[77] Uchida G., Takenaka K., Setsuhara Y. (2015). Effects of discharge voltage waveform on the discharge characteristics in a helium atmospheric plasma jet. J. Appl. Phys..

[78] Fridman A., Kennedy L.A. (2011). Plasma Physics and Engineering.

[79] Rumbach P., Witzke M., Sankaran R.M., Go D.B., Edu D. Plasma-liquid interactions: Separating electrolytic reactions from plasma/gas phase reactions. Proceedings of the ESA Annual Meeting on Electrostatics.

[80] Hamdan A., Profili J., Cha M.S. (2020). Microwave Plasma Jet in Water: Effect of Water Electrical Conductivity on Plasma Characteristics. Plasma Chem. Plasma Process..

[81] Vanraes P., Bogaerts A. (2018). Plasma physics of liquids—A focused review. Appl. Phys. Rev..

[82] Yoon S.Y., Jeon H., Yi C., Park S., Ryu S., Kim S.B. (2018). Mutual Interaction between Plasma Characteristics and Liquid Properties in AC-driven Pin-to-Liquid Discharge. Sci. Rep..

[83] Misyura S.Y. (2017). Evaporation of a sessile water drop and a drop of aqueous salt solution. Sci. Rep..

[84] Maguire P.D., Mahony C.M.O., Kelsey C.P., Bingham A.J., Montgomery E.P., Bennet E.D., Potts H.E., Rutherford D.C.E., McDowell D.A., Diver D.A. (2015). Controlled microdroplet transport in an atmospheric pressure microplasma. Appl. Phys. Lett..

[85] Stancampiano A., Bocanegra P.E., Dozias S., Pouvesle J.M., Robert E. (2021). Evidence, origin and impact of liquid flows in plasma medicine in vitro treatments with APPJs. Plasma Sources Sci. Technol..

